# Supplementary material for: Heat adaptation of phage T7 under an extended genetic code
Source: Virus Evol. 2021 Dec 1;7(2):veab100. doi: 10.1093/ve/veab100 (PMC8923235; doi:10.1093/ve/veab100)
Supplement: veab100_Supp [file veab100_supp.zip › Sequencing_Analysis.zip!/Sequencing_Analysis/A1/evidence/JC_53.html]

BRESEQ :: Evidence


| New junction evidence | | | | | | | | | | | |
| --- | --- | --- | --- | --- | --- | --- | --- | --- | --- | --- | --- |
|  | | seq id | position | reads (cov) | reads (cov) | score | skew | freq | annotation | gene | product |
| \* | ? | NC\_001604.1 | = 9789 | 3293 (1.200) | 306 (0.110) +CGACGAAGAAAGCTGGGACGAAGA | 4/486 | 42.4 | 8.3% | intergenic (–/–) | *–/–* | –/– |
|  | ? | NC\_001604.1 | 9790 = | 3613 (1.170) |  |  |  |  | intergenic (–/–) | *–/–* | –/– |
| Rejected: Coverage evenness skew score above cutoff. | | | | | | | | | | | |
| Rejected: Frequency below/above cutoff threshold. | | | | | | | | | | | |

Only 100 of 307 total aligned reads displayed.  

`GCGTCTTTCCAAGACAAGAAGACCAAAGAGACC‑AAGCACATCAATCTGGTTGTGGTTGACTCAA‑‑‑‑‑AAGGTAAGAAGATGGAAGACGTTCCGATTATCGGTGGTGGCTCTAAGCTGAAAGTTAAATATTCTCTGGTTCCATACAAGTGGAACACTGCTGTAGGTGCGAGCGTTAAGCTGCAACTGGAATCCGTGATGCTGGTCGAACTGGCTACCTTTGGTGGCGGTGAAGACGATTGGGCTGACGAAGTTGAAGAGAACGGCTATGTTGCCTCTGGTTCTGCCAAAGCGAGCAAACCACG‑‑‑‑‑‑‑‑‑‑‑‑‑‑‑‑‑‑‑‑‑‑‑‑‑‑‑‑‑‑‑‑‑‑‑‑‑‑‑‑‑‑‑‑‑‑‑‑‑‑‑‑‑‑‑‑‑‑‑‑‑‑‑‑‑‑‑‑‑‑‑‑‑‑‑‑‑‑‑‑‑‑‑‑‑‑‑‑‑‑‑‑‑‑‑‑‑‑‑‑‑‑‑‑‑‑‑‑‑‑‑‑‑‑‑‑‑‑‑‑‑‑‑‑‑‑‑‑‑‑‑‑‑‑‑‑‑‑‑‑‑‑‑‑‑‑‑‑‑‑‑‑‑‑‑‑‑‑‑‑‑‑‑‑‑‑‑‑‑‑‑‑‑‑‑‑‑‑‑‑‑‑‑‑‑‑‑‑‑‑‑‑‑‑‑‑‑‑‑‑‑‑‑‑‑‑‑‑‑‑‑‑‑‑‑‑‑‑‑‑‑‑‑‑‑‑‑‑‑‑‑‑‑‑‑‑‑‑‑‑‑‑‑‑‑‑‑‑‑‑‑‑‑‑‑‑‑‑‑‑‑‑‑‑‑‑‑‑‑‑‑‑‑‑‑‑‑‑‑‑‑‑‑‑‑‑‑‑‑‑‑‑‑‑‑‑‑‑‑‑‑‑‑‑‑‑  >  NC_001604.1/9491‑9789`  
`‑‑‑‑‑‑‑‑‑‑‑‑‑‑‑‑‑‑‑‑‑‑‑‑‑‑‑‑‑‑‑‑‑‑‑‑‑‑‑‑‑‑‑‑‑‑‑‑‑‑‑‑‑‑‑‑‑‑‑‑‑‑‑‑‑‑‑‑‑‑‑‑‑‑‑‑‑‑‑‑‑‑‑‑‑‑‑‑‑‑‑‑‑‑‑‑‑‑‑‑‑‑‑‑‑‑‑‑‑‑‑‑‑‑‑‑‑‑‑‑‑‑‑‑‑‑‑‑‑‑‑‑‑‑‑‑‑‑‑‑‑‑‑‑‑‑‑‑‑‑‑‑‑‑‑‑‑‑‑‑‑‑‑‑‑‑‑‑‑‑‑‑‑‑‑‑‑‑‑‑‑‑‑‑‑‑‑‑‑‑‑‑‑‑‑‑‑‑‑‑‑‑‑‑‑‑‑‑‑‑‑‑‑‑‑‑‑‑‑‑‑‑‑‑‑‑‑‑‑‑‑‑‑‑‑‑‑‑‑‑‑‑‑‑‑‑‑‑‑‑‑‑‑‑‑‑‑‑‑‑‑‑‑‑‑‑‑‑‑‑‑‑‑‑‑‑‑‑‑‑‑‑‑‑‑‑‑‑‑‑‑‑‑‑‑‑‑‑‑‑‑‑‑‑‑‑‑‑‑‑‑‑‑‑‑‑‑‑‑‑‑‑‑‑‑‑‑‑‑‑CGACGAAGAAAGCTGGGACGAAGACGACGAAGAGTCCGAGGAAGCAGACGAAGACGGAGACTTCTAAGTGGAACTGCGGGAGAAAATCCTTGAGCGAATCAAGGTGACTTCCTCTGGGTGTTGGGAGTGGCAGGGCGCTACGAACAATAAAGGGTACGGGCAGGTGTGGTGCAGCAATACCGGAAAGGTTGTCTACTGTCATCGCGTAATGTCTAATGCTCCGAAAGGTTCTACCGTCCTGCACTCCTGTGATAATCCATTATGTTGTAACCCTGAACACC  >  NC_001604.1/9790‑10070`  

                                                                                                                                                                                                                                                                                                                 `|||||||||||||||||||||||||`                                                                                                                                                                                                                                                                                           
 `CGTCTTTCCAAGACAAGAAGACCAAAGAGACC‑AAGCACATCAATCTGGTTGTGGTTGACTCAA‑‑‑‑‑AAGGTAAGAAGATGGAAGACGTTCCGATTATCGGTGGTGGCTATAAGCTGAAAGTTAAATATTCTCTGGTTCCATACAAGTTGAACACTGCTGTAGGTGCGAGCGTTAAGCTGCAACTGGAATCCGGGATGCTGGTCGAACTGGCTACCTTTGGTGGCGGTGAAGACGATTGGGCTGACGAAGTTGAAGAGAACGGCTATGTTGCCTCTGGTTCTGCCAAAGCGAGCAAACCACGCGA                                                                                                                                                                                                                                                                                                                 >  1:326308/1‑301`  
 `CGTCTTGCAAAGAAAAGAAGACCAAAGAGACC‑AAGCACATCAATCTGGTTGTGGTTGACTCAA‑‑‑‑‑AAGGGAAGAAGATGGAAGACGTTCCGATTATCGGTGGTGGCTCTAAGCTGAAAGTTAAATAGTCTCTGGTTCCAGACAAGTGGAACACGGCTGTAGGTGCGAGTGTTAAGCTGCAACTGGAATCCGGGATGCTGGTCGAACTGGCTACCTTTGGTGGCGGTGAAGACGATTGGGCTGACGAAGTTGAAGAGAACGGCTATGTTGCCTCTGGTTCTGCCAAAGCGAGCAAACCACGCGA                                                                                                                                                                                                                                                                                                                 <  1:451626/301‑1`  
 `CGTCGTTCCAAGACAAGAAGACCAAAGAGACC‑AAGCACATCAATCTGGTTGTGGTTGACTCAA‑‑‑‑‑AAGGTAAGAAGATGGAAGACGTTCCGATTATCGGTGGTGGCTCTAAGCTGAAAGTTAAATATTCTCTGGTTCCATACAAGTGGAACACTGCTGTAGGTGCGAGCGTTAAGCTGCAACTGGAATCCGTGATGCTGGTCGAACTGGCTACCTTTGGTGGCGGTGAAGACGATTGGGCTGACGAAGTTGAAGAGAACGGCTATGTTGCCTCTGGTTCTGCCAAAGCGAGCAAACCACGCGA                                                                                                                                                                                                                                                                                                                 <  1:450877/301‑1`  
  `GTCTTTCCAAGACAAGAAGACCAAAGAGGCC‑AAGCACATCAATCGGGTTGTGGTTGACTCAA‑‑‑‑‑AAGGTAAGAAGATGGAAGACGTTCCGATTATCGGTGGTGGCTCTAAGCTGAAAGTTAAATATTCTCTGGTTCCATACAAGTGGAACGCTGCTGTAGGTGCGAGCGTTAAGCTGCAACTGGAATCCGTGATGCTGGTCGAACTGGCTACCTTTGGTGGCGGTGAAGACGATTGGGCTGACGAAGTTGAAGAGAACGGCTATGTTGCCTCTGGTTCTGCCAAAGCGAGCAAACCACGCGAC                                                                                                                                                                                                                                                                                                                <  1:111906/301‑1`  
   `TCTTTCCAAGACCAGAAGACCAAAGAGACC‑AAGCACATCAATCTGGTTGTGGTTGACTCAA‑‑‑‑‑AAGGTAAGAAGATGGAAGACGTTCCGATTATCGGTGGTGGCTCTAAGCTGAAAGTTAAATATTCTCTGGTTCCATACAAGTGGAACACTGCTGTAGGTGCGAGCGTTAAGATGCAACTGGAATCCGTGATGCTGGTCGAACTGGCTACCTTTGGTGGCGGTGAAGACGATTGGGCTGACGAAGTTGAAGAGAACGGCTATGTTGCCTCTGGTTCTGCCAAAGCGAGCAAACCACGCGACG                                                                                                                                                                                                                                                                                                               >  1:409586/1‑301`  
   `TCTTTCCAAGACAAGAAGACCAAAGAGACC‑AAGCACATCAATCTGGTTGTGGTTGACTCAA‑‑‑‑‑AAGGTAAGAAGATGGAAGACGTTCCGATTATCGGTGGTGGCTCTAAGCTGAAAGTTAAATATTCTCTGGTTCCATACAAGTGGAACACTGCTGTAGGTGCGAGCGTTAAGCTGCAACTGGAATCCGTGATGCTGGTCGAACTGGCTACCTTTGGTGGCGGTGAAGACGATTGGGCTGACGAAGTTGAAGAGAACGGCTATGTTGCCTCTGGTTCTGCCAAAGCGAGCAGACCACGCGACG                                                                                                                                                                                                                                                                                                               >  1:395299/1‑301`  
      `TTCCAAGACAAGAAGACCAAAGAGACC‑AAGCACATCAATCTGGTTGTGGTTGACTCAA‑‑‑‑‑AAGGTAAGAAGATGGAAGACGTTCCGATTATCGGTGGTGGCTCTAAGCTGAAAGTTAAATATTCTCTGGTTCCATACAAGTGGAACACTGCTGTAGGTGCGAGCGTTAAGCTGCAACTGGAATCCGTGATGCTGTTCGAACTGGATACCTTTGGTGGCGGTGAAGACGCTTGGGCTGACGAAGTTGAAGAGAACGGCTATGTTGCCTCTGGTTCTGCAAAAGCGAGCAAACCACGCGACGAAG                                                                                                                                                                                                                                                                                                            >  1:103376/1‑301`  
      `TTCCAAGACAAGAAGACCAAAGAGACC‑AAGCACATCAATCTGGTTGTGGTTGACTCAA‑‑‑‑‑AAGGTAAGAAGATGGAAGACGTTCCGATTATCGGTGGTGGCTCTAAGCTGAAAGTTAAATATTCTCTGGTTCCATACAAGTGGAACACTGCTGTAGGTGCGAGCGTTAAGCTGCAACTGGAATCCGTGATGCTGGTCGAACTGGCTACCTTTGGTGGCGGTGAAGACGATTGGGCTGACGACGTTGAAGAGAACGGCTATGTTGCCTCTGGTTCTGCCAAAGCGAGCAAACCACGCCACGAAa                                                                                                                                                                                                                                                                                                            >  1:354573/1‑300`  
      `TTCCAAGACAAGAAGACCAAAGAGACC‑AAGCACATCAATCTGGTTGTGGTTGACTCAA‑‑‑‑‑AAGGTAAGAAGATGGAAGACGTTCCGATTATCGGTGGTGGCTCTAAGCTGAAAGTTAAATATTCTCTGGTTCCATACAAGTGGAACACTGCTGTAGGTGCGAGCGTTAAGCTGCAACTGGAATCCGTGATGCTGGTCGAACTGGCTACCTTTGGTGGCGGTGAAGACGATTGGGCTGACGAAGTTGAAGAGAACGGCTATGTTGCCTCTGGTTCTGCCAAAGCGAGCAAACCACGCGACGAAG                                                                                                                                                                                                                                                                                                            >  1:246381/1‑301`  
      `TTCCAAGACAAGAAGACCAAAGAGACC‑AAGCACATCAATCTGGTTGTGGTTGACTCAA‑‑‑‑‑AAGGTAAGAAGATGGAAGACGTTCCGATTATCGGTGGTGGCTCTAAGCTGAAAGTTAAATATTCTCTGGTTCCATACAAGTGGAACACTGCTGTAGGTGCGAGCGTTAAGCTGCAACTGGAATCCGTGATGCTGGTCGAACTGGCTACCTTTGGTGGCGGTGAAGACGATTGGGCTGACGAAGTTGAAGAGAACGGCTATGTTGCCTCTGATTCTGCCAAAGCGAGCAAACCACGCGACGAAG                                                                                                                                                                                                                                                                                                            >  1:313069/1‑301`  
      `TTCCAAGACAAGAAGACCAAAGAGACC‑AAGCACATCAATCTGGTTGTGGTTGACTCAA‑‑‑‑‑AAGGTAAGAAGATGGAAGACGTTCCGATTATCGGTGGTGGCTCTAAGCTGAAAGTTAAATATTCTCTGGTTCCATACAAGTGGAACACTGCTGTAGGTGCGAGCGTTAAGCTGCAACTGGAATCCGTGATGCTGGTCGAACTGGCTACCTTTGGTGGCGGTGAAGACGATTGGGCTGACGAAGTTGAAGAGAACGACTATGTTGCCTCTGGTTCTGCCAAAGCGAGCAAACCACGCGACGAAG                                                                                                                                                                                                                                                                                                            >  1:161044/1‑301`  
         `agatgtgtataagagacAGAGACC‑AAGCACATCAATCTGGTTGTGGTTGACTCAA‑‑‑‑‑AAGGTAAGAAGATGGAAGACGTTCCGATTATCGGTGGTGGCTCTAAGCTGAAAGTTAAATATTCTCTGGTTCCATACAAGTGGAACACTGCTGTAGGTGCGAGCGTTAAGCTGCAACTGGAATCCGTGATGCTGGTCGAACTGGCTACCTTTGGTGGCGGTGAAGACGATTGGGCTGACGAAGTTGAAGAGAACGGCTATGTTGCCTCTGGTTCTGCCAAAGCGAGCAAACCACGCGACGAAGAAA                                                                                                                                                                                                                                                                                                         <  1:344452/284‑1`  
         `CAAGACAAGAAGACCAAAGAGACC‑AAGCACATCAATCTGGTTGTGGTTGACTCAA‑‑‑‑‑AAGGTAAGAAGATGGAAGACGTTCCGATTATCGGTGGTGGATCTAAGCTGAAAGTTAAATATTCTCTGGTTCCATACAAGTGGAACACTGCTGTAGGTGCGAGCGTTAAGCTGCAACTGGAATCCGTGATGCTGGTCGAACTGGCTACCTTTGGTGGCGGTGAAGACGATTGGGCTGACGAAGTTGAAGAGAACGGCTATGTTGCCTCTGGTTCTGCCAAAGCGAGCAAACCACGCGACAAAGAct                                                                                                                                                                                                                                                                                                         >  1:38670/1‑299`  
           `aagagacagGACCAAAGAGACC‑AAGCACATCAATCTGGTTGTGGTTGACTCAA‑‑‑‑‑AAGGTAAGAAGATGGAAGACGTTCCGATTATCGGTGGTGGCTCTAAGCTGAAAGTTAAATATTCTCTGGTTCCATACAAGTGGAACACTGCTGTAGGTGCGAGCGTTAAGCTGCAACTGGAATCCGTGATGCTGGTCGAACTGGCTACCTTTGGTGGCGGTGAAGACGATTGGGCTGACGAAGTTGAAGAGAACGGCTATGTTGCCTCTGGTTCTGCCAAAGCGAGCAAACCACGCGACGAAGAAAGC                                                                                                                                                                                                                                                                                                       <  1:23596/292‑1`  
           `aaagagacAGACCAAAGAGACC‑AAGCACATCAATCTGGTTGTGGTTGACTCAA‑‑‑‑‑AAGGTAAGAAGATGGAAGACGTTCCGATTATCGGTGGTGGCTCTAAGATGAAAGTTAAATATTCTCTGGTTCCATACAAGTGGAACACTGCTGTAGGTGCGAGCGTTAAGCTGCAACTGGAATCCGTGATGCTGGTCGAACTGGCTACCTTTGGTGGCGGTGAAGACGATTGGGCTGACGAAGTTGAAGAGAACGGCTATGTTGCCTCTGGTTCTGCCAAAGCGAGCAAACCACGCGACGAAGAAAGC                                                                                                                                                                                                                                                                                                       <  1:502774/293‑1`  
           `AGACAAGAAGACCAAAGAGACC‑AAGCACATCAATCTGGTTGTGGTTGACTCAA‑‑‑‑‑AAGGTAAGAAGATGGAAGACGTTCCGATTATCGGTTGTGGCTCTAAGCTGAAAGTTAAATATTCTCTGGTTCCATACAAGTGGAACACTGCTGTAGGTGCGAGCGTTAAGCTGCAACTGGAATCCGTGATGCTGGTCGAACTGGCTACCTTTGGTGGCGGTGAAGACGATTGGGCTGACGAAGTTGAAGAGAACGGCTATGTTGCCTCTGGTTCTGCCAAAGCGAGCAAACCACGCGACGAAGAAAGC                                                                                                                                                                                                                                                                                                       <  1:29928/301‑1`  
           `AGAAAAAAAGACAAAATAGACC‑AAGCACAGCAATGTGGTTGTGGATGACTCAA‑‑‑‑‑AAGGTAAGAAAAGGGAAGACGTTCCGATTATCGGTGGTGGCTCTAAGCTGAAAGTTAAATATTCACTGGTTCCATACAAGTGGAACACTGCTGTAGGTGCTAGCGTTAAGCTGCAACTGGAATCCGGGATGCTGGTCGAACTGGCTACCTTTGGTGGCGGTGAAGACGATTGGGCTGACGAAGTTGAAGAGAACGGCTATGTTGCCTCTGGTTCTGCCAAAGCGAGCAAACCCCGCGACGAAGAAAGC                                                                                                                                                                                                                                                                                                       <  1:490510/301‑1`  
            `gggctcggagatttgtataagagacagCATCAATCTGGTTGTGGTTGACTCAA‑‑‑‑‑AAGGTAAGAAGATGGAAGACGTTCCGATTATCGGTGGTGACTCTAAGCTGAAAGTTAAATATTCTCTGGTTCCATACAAGTGGAACACTGCTGTAGGTGCGAGCGTTAAGCTGCAACTGGAATCCGTGATGCTGGTCGAACTGGCTACCTTTGGTGGCGGTGAAGACGATTGGGCTGACGAAGTTGAAGAGAACGGCTATGTTGCCTCTGGTTCTGCCAAAGCGAGCAAACCACGCGACGAAGAAAGC                                                                                                                                                                                                                                                                                                       <  1:385066/274‑1`  
            `GACAAGAAGACCAAAGAGACC‑AAGCACATCAATCTGGTTGTGGTTGACTCAA‑‑‑‑‑AAGGTAAGAAGATGGAAGACGTTCCGATTATCGGTGGTGGCTCTAAGCTGAAAGTTAAATATTCTCTGGTTCCATACAAGTGGAACACTGCTGTATGTGCGAGCGTTAAGCTGCAACTGGAATCCGTGATGCTGGTCGAACTGGCTACCTTTGGTGGCGGTGAAGACGATTGGGCTGACGAAGTTGAAGAGAACGGCGATGTTGCCTCTGGTTCTGCCAAAGCGAGCAAACCACTCGACGAAGAAAGCT                                                                                                                                                                                                                                                                                                      >  1:60378/1‑301`  
            `GACAAGAAGACCAAAGAGACC‑AAGCACATCAATCTGGTTGTGGTTGACTCAA‑‑‑‑‑AAGGTAAGAAGATGGAAGACGTTCCGATTATCGGTGGTGGCTCTAAGCTGAAAGTTAAATATTCTCTGGTTCCATACAAGTGGAACACTGCTGTAGGTGCGAGCGTTAAGCTGCAACTGGAATCCGTGATGCTGGTCGAACTGGCTACCTTTGGTGGCGGTGAAGACGATTGGGCTGACGAAGTTGAAGAGAACGGCTATGTTGCCTCTGGTTCTGCCAAAGCGAGCAAACCCCGCGACGAAGAAAGCc                                                                                                                                                                                                                                                                                                      >  1:278793/1‑300`  
            `GACAAGAAGACCAAAGAGACC‑AAGCACATCAATCTGGTTGTGGTTGACTCAA‑‑‑‑‑AAGGTAAGAAGATGGAAGACGTTCCGATTATCGGTGGTGGCTCTAAGCTGAAAGTTAAATATTCTCTGGTTCCATACAAGTGGAACACTGCTGTAGGTGCGAGCGTTAAGCTGCAACTGGAATCCGTGATGCTGGTCGAACTGGCTACCTTTGGTGGCGGTGAAGACGATTGGGCTGACGAAGTTGAAGAGAACGGCTATGTTGCCTCTGGTTCTGCCAAAGCGAGCAAACCACGCGACGAAGAAAGCT                                                                                                                                                                                                                                                                                                      >  1:341997/1‑301`  
            `GACAAGAAGACCAAAGAGACC‑AAGCACATCAATCTGGTTGTGGTTGACTCAA‑‑‑‑‑AAGGTAAGAAGATGGAAGACGTTCCGATTATCGGTGGTGGCTCTAAGCTGAAAGTTAAATATTCTCTGGTTCCATACAAGTGGAACACTGCTGTAGGTGCGAGCGTTAAGCTGCAACTGGAATCCGTGATGCTGGTCGAACTGGCTACCTTTGGTGGCGGTGAAGACGATTGGGCTGACGAAGTTGAAGAGAACGGCTATGTTGCCTCTGGTTCTGCAAAAGCGAGCAAACCACGCGACGAAGAAAGCg                                                                                                                                                                                                                                                                                                      >  1:229163/1‑300`  
            `GACAAGAAGACCAAAGAGACC‑AAGCACATCAATCTGGTTGTGGTTGACTCAA‑‑‑‑‑AAGGTAAGAAGATGGAAGACGTTCCGATTATCGGTGGTGGCTCTAAGCTGAAAGTTAAAGATTCTCTGGTTCCATACAAGTGGAACACTGCTGTAGGTGCGATCGTTAAGCTGCAACTGGAATCCGTGATGCTGGTCGAACTGGCTACCTTTGGTGGCGGTGAAGACGATTGGGCTGACGAAGTTGAAGAGAACGGCTATGTTGCCTCTGGTTCTGCCAAAGCGAGCAAACCACGCGACGAAGAAAGCg                                                                                                                                                                                                                                                                                                      >  1:244943/1‑300`  
            `GACAAGAAGACCAAAGAGACC‑AAGCACATCAATCTGGTTGTGGTTGACTCAA‑‑‑‑‑AAGGTAAGAAGATGGAAGACGTTCCGATTATCGGTGGTGGCTCTAAGCTGAAAGTGAAATATTCTCTGGTTCCATACAAGTGGAACACTGCTGTAGGTGCGAGCGTTAAGCTGCAACTGGAATCCGTGATGCTGGTCGAACTGGCTACCTTTGGTGGCGGTTAAGACGATTGGGCTGACGAAGTTGAAGAGAACGGCTATGTTGCCTCTGGTTCTGCCAAAGCGAGCAAACCACGTGACGAAGAAAGCT                                                                                                                                                                                                                                                                                                      >  1:51746/1‑301`  
             `agacagAGACCAAAGAGACC‑AAGCACATCAATCTGGTTGTGGTTGACTCAA‑‑‑‑‑AAGGTAAGAAGATGGAAGACGTTCCGATTATCGGTGGTGGCTCTAAGCTGAAAGTTAAATATTCTCTGGTTCCATACAAGTGGAACACTGCTGTAGGTGCGAGCGTTAAGCTGCAACTGGAATCCGTGATGCTTGTCGAACTGGCTACATTTGGTGGCGGTGAAGACGATTGGGCTGACGAAGTTGAAGAGAACGGCTATGTTGCCTCTGGTTCTGCCAAAGCGAGCAAACCACGCGACGAAGAAAGCTG                                                                                                                                                                                                                                                                                                     <  1:131943/295‑1`  
              `aAAGAAGACCGAAGAGACC‑AAGCACATCAATCTGGTTGTAGTTGACTCAA‑‑‑‑‑AAAGTAAGAAGATGGAAGACGTTCCGATTATCGGTGGTGGCTCTAAGCTGAAAGTTAAATATTCTCTGGCTCCATACAAGTGGAACACTGCTGTAGGTGCGAGCGTTAAGCTGTAACTGGAATCCGTGATGCTGGTCGAACTGGCTACCTTTGGTGGCGGTGAAGACGATTGGGCTGACGAAGTTGAAGAGAACGGCTATGTTGCCTCTGGTTCTGCCAAAGCGAGCAAACCACGCGACGAAGAAAGCTGG                                                                                                                                                                                                                                                                                                    <  1:234280/300‑1`  
              `aAAGAAGACCAAAGAGACC‑AAGCACAGCGATCTGGTTGTGGTTGACTCAA‑‑‑‑‑AAGGTAAGAAGATGGAAGACGTTCCGGTTATCGGTGGTGGCTCTAAGCTGAAAGTTAAATATTCTCTGGTTCCATACAAGGGGAACACTGCTGGAGGTGCGAGCGTTAAGCTGCAACTGGAATCCGTGATGCTGGTCGAACTGGCTACCTTTGGTGGCGGTGAAGACGATTGGGCTGACGAAGTTGAAGAGAACGGCTATGTTGCCTCTGGTTCTGCCAAAGCGAGCAAACCACGCGACGAAGAAAGCTGG                                                                                                                                                                                                                                                                                                    <  1:87054/300‑1`  
              `CAAGAAGACCAAAGAGACC‑AAGCACATCAATCTGGTTGTGGTTGACTCAA‑‑‑‑‑AAGGTAAGAAGATGGAAGACGTTCCGATTATCGGTGGTGGCTCTAAGCTGAAAGTTAAATATTCTCTGGTTCCATACAAGTGGAACACTGCTGTAGGTGCGAGCGTTAAGCTGCAACTGGAATCCGTGATGCTGGTCGAACTGGCTACCTTTGGTGGCGGTGAAGACGATTGGGCTGACGAAGTTGAAGAGAACGGCTATGTTGCCTCTGGTTCTGCCAAAGCGAGCAAACCACGCGACGAAGAAAGCAGG                                                                                                                                                                                                                                                                                                    >  1:196498/1‑301`  
               `AAGAAGACCAAAGAGACC‑AAGCAAATCAATCTGGTTGTGGTTGACCAAA‑‑‑‑‑AAGGTAAGAAGATGGAAGACGTTCCTATTATCGGTGGTGGCTCTAAGCTGAAAGTTAAATATTCTCTGGTTCCATACAAGTGGAACACTGCTGTAGGTGCGAGCGTTAAGCTGCAACTGGAATCCGTGATGCTGGTCGAACTGGCTACCTTTGGTGGCGGTGAAGACGATTGGGCTGACGAAGTTGAAGAGAACGGCTATGTTGCCTCTGGTTCTGCCAAAGCGAGCAAACCACGCGACGAAGAAAGCTGGG                                                                                                                                                                                                                                                                                                   <  1:372991/301‑1`  
                `AGAAGACCAAATAGACC‑AATCGCATCAATCTGGTCGTGGTTGACTCAA‑‑‑‑‑AAGGTAAGAAGATGGAAGTCGTTCCTATTATCGGTGGTGGCTCTAAGCTGAAAGTTAAATATTCTCTGGTTCCATACAAGTGGAACACTGCTGTAGGTGCGAGCGTTAAGCTGCAACTGGAATCCGTGATGCTGGTCGAACTGACTACCTTTGGTGGCGGTTAAGACGATTGGGCTGACGAAGTTGAAGAGAACGGCTATGTTGCCTCTGGTTCTGCCAAAGCGAGCAACCCACGCGACGAAGAAAGCTGGGA                                                                                                                                                                                                                                                                                                  <  1:461627/301‑1`  
                `AGAAGACCAAAGAGACC‑AAGCACATCAATCTGGTTGTGGTTGACTCAA‑‑‑‑‑AAGGTAAGAAGATGGAAGACGTTCCGATTATCGGTGGTGGCTCTAAGCTGAAAGTTAAATATTCTCTGGTTCCATACAAGTGGAACACTGCTGTAGGTGCGAGCGTTAAGCTGCAACTGGAATCCGTGATGCTGGTCGAACTGGCTACCTTTGGTGGCGGTGAAGACGATTGGGCTGACGAAGTTGAAGAGAACGGCTATGTTGCCTCTGGTTCTGCCAAAGCGAGCAAACCACGCGACGAAGAAAGCTGGGA                                                                                                                                                                                                                                                                                                  >  1:177290/1‑301`  
                `AGAAGACCAAAGAGACC‑AAGCACATCAATCTGGTTGTGGTTGACTCAA‑‑‑‑‑AAGGTAAGAAGATGGAAGACGTTCCGATTATCGGTGGTGGCTCTAAGCTGAAAGTTAAATATTCTCTGGTTCCATACAAGTGGAACACTGCTGTAGGTGCGAGCGTTAAGCTGCAACTGGAATCCGTGATGCTGGTCGAACTGGCTACCTTTGGTGGCGGTGAAGACGATTGGGCTGACGAAGTTGAAGAGAACGGCTATGTTGCCTCTGGTTCTGCCAAAGCGAGCAAACCACGCGACGAAGAAAGCTGGGA                                                                                                                                                                                                                                                                                                  <  1:359957/301‑1`  
                `AGAAGACCAAAGAGACC‑AAGCACATCAATCTGGTTGTGGTTGACTCAA‑‑‑‑‑AAGGGAAGAAGATGGAAGACGTTCCGATTATCGGTGGTGGCTCTAAGCTGAAAGTTAAATATTCTCTGGTTCAATACAAGTGGAACACTGCTGTAGGTGCGAGCGTTAAGCTGCAACTGGAATCCGTGATGCTGGTCGAACTGGCTACCTTTGGTGGCGGTGAAGACGATTGGGCTGACGAAGTTGAAGAGAACGGCTATGTTGCCTCTGGTTCTGCCAAAGCGAGCAAACCACGCGACGAAGAAAGCTGGGA                                                                                                                                                                                                                                                                                                  <  1:173713/301‑1`  
                 `GAAGATCAAAGAGACC‑AAGCACATCAATCTGGTTGTGGTTGACTCAA‑‑‑‑‑AAGGTAAGAAGATGGAAGACGTTCCGATTATCGGTGGTGGCTCTAAGCTGAAAGTTAAATATTCTCTGGTTCCATACAAGTGGAACACTGCTGTAGGTTCGAGCGTTAAGCTGCAACTGGAATCCGTGATGCTGGTCTAACTGGCTATCTTTGGTGGCGGTGAAGACGATTGGGCTGACGAAGTTGAAGAGAACGGCTATGTTGCCTCTGGTTCTGCCAAATCGAGCAAACCACGCGACGAAGAAAGCTGGGAC                                                                                                                                                                                                                                                                                                 >  1:274211/1‑301`  
                 `GAAGACCAAAGATACC‑AAGCACATCAATCTGGTTGTGGTTGACTCAA‑‑‑‑‑AAGGTAAGAAGATGGAAGACGTTCCGATTATCGGTGGTGGCTCTAAGCTGAAAGTTAAATATTCTCTGGTTCCATACAAGTGGAACACTGCTGTAGGTGCGAGCGTTAAGCTGCAACTGGAATCCGTGATGCTGGTCGAACTGGCTACCTTTGGTGGCGATGAAGACGATTGGGCTGACGAAGTTGAAGAGAACGGCTATGTTGCCTCTGGTTCTGCCAAAGCGAGCAAACCACGCGACGAAGAAAGCTGGGAC                                                                                                                                                                                                                                                                                                 >  1:284932/1‑301`  
                 `GAAGACCAAAGAGACC‑AAGCACATCAATCTGGTTGTGGTTGACTCAA‑‑‑‑‑AAGGTAAGAAGATGGAAGACGTTCCGATTATCGGTGGTGGCTCTAAGCTGAAAGTTAAATATTCTCTGGTTCCATACAAGTGGAACACTGCTGTAGGTGCGAGAGTTAAGCTGCAACTGGAATCCGTGATGCTGGTCGAACTGGCTACCTTTGGTGGCGGTGAAGACGATTGGGCTGACGAAGTTGAAGAGAACGGCTATGTTGCCTCTGGTTCTGCCAAAGCGAGCAAACCACGCGACGAAGAAAGCTGGGAC                                                                                                                                                                                                                                                                                                 >  1:55596/1‑301`  
                  `acagCCAAAGAGACC‑AATGACATCAATCTGGTTGTGGTTGACTCAA‑‑‑‑‑AAGGTAAGAAGATGGAAGACGTTCCGATTATCGGTTGTGGCTCTAAGCTGAAAGTTAAATATTCTCTGGTTCCATACAAGTGGAACACTGCTGTAGGTGCGAGCGTTAAGCTGCAACTGGAATCCGTGATGCTGGTCGAACTGGCTACCTTTGGTGGCGGTGAAGACGATTGGGCTGACGAAGTTGAAGAGAACGGCTATGTTGCCTCTGGTTCTGCCAAAGCGAGCAAACCACGCGACGAAGAAAGCTGGGAC‑G                                                                                                                                                                                                                                                                                               <  1:44339/297‑1`  
                  `AAGACAAAAGAGACC‑AAGCAAATCAATCTGGTTGTGGTTGACTCAA‑‑‑‑‑AAGGTAAGAAGATGGAAGACGTTCCGATTATCGGTGGTGGCTCTAAGCTGAAAGTTAAATATTCTGTGGTTCCATGCAAGTGGAACACTGCTGTAGGTGCGAGCGTTAAGCTGAAACTGGAATCCGTGATGCTGGTCGAACTGGCTACCTTTGGTGGCGGTGAAGACGATTGGGCTGACGAAGTTGAAGAGAACGGCTATGTTGCCTCTGGTTCTGCCAAAGCGAGCAAACCACGCGACGAAGAAAGCTGGGAC‑G                                                                                                                                                                                                                                                                                               <  1:436657/301‑1`  
                   `AGACCAAAGAGACC‑AAGCACATCAATCTGGTTGTGGTTGCCTAAA‑‑‑‑‑TCGGTAAGAAGATGGAAGACGTTCCGATTATCGGTGGTGGCTCTAAGCTGAAAGTTAAATATTCTCTGGTTCCATCCAAGTGGAACACCGCTGTAGGTGCGAGCGTTAAGCTGCCACTGGAATCCGTGATGCCTGTCTAACTGGCTACCTTTTGTGGCGGTGAAGACGATTGGGCTGACGAAGTTGAAGAGAACGGCTATGTTGCCTCTCGTTCTGCCAAAGCGAGCACACCACGCGACGAAGAAAGCTGGGACtg                                                                                                                                                                                                                                                                                               >  1:491323/1‑299`  
                   `AGACCAAAGAGACC‑AAGCACATCAATCTGGTTGTGGTTGACTCAAAAGGTAAGGTAAGAAGATGGAAGACGTTCCGATTATCGGTGGTGGCTCTAAGCTGAAAGTTAAATATTCTCTGGTTCCATACAAGTGGAACACTGCTGTAGGTGCGAGCGTTAAGCTGCAACTGGAATCCGTGATGCTGGTCGAACTGGCTACCTTTGGTGGCGGTGAAGACGATTGGGCTGACGAAGTTGAAGAGAACGGCTATGTTGCCTCTGGTTCTGCCAAAGCGAGCAAACCACGCGACGAAGAAAGCTGG                                                                                                                                                                                                                                                                                                    >  1:469675/1‑301`  
                   `AGACCAAAGAGACC‑AAGCACATCAATCTGGTTGTGGTTGACTCAA‑‑‑‑‑AAGGTAAGAAGATGGAAGACGTTCCGATTATCGGTGGTGGCTCTAAGCTGAAAGTTAAATATTCTCTGGTTCCATACAAGTGGAACACTGCTGTAGGTGCGAGCGTTAAGCTGCAACTGGAATCCGTGATGCTGGTCGAACTGGCTACCTTTGGTGGCGGTGAAGACGATTGGGCTGACGAAGTTGAAGAGAACGGCTATGTTGCCTCTGGTTCTGCCAAAGCGAGCAAACCACGCGACGAAGAAAGCTGGGctgt                                                                                                                                                                                                                                                                                               >  1:419678/1‑297`  
                   `AGACCAAAGAGACC‑AAGCACATCAATCTGGTTGTGGTTGACTCAA‑‑‑‑‑AAGGTAAGAAGATGGAAGACGTTCCGATTATCGGTGGTGGCTCTAAGCTGAAAGTTAAATATTCTCTGGTTCCATACAAGTGGAACACTGCTGTAGGTGCGAGCGTTAAGCTGCAACTGGAATCCGTGATGCTGGTCGAACTGGCTACCTTTGGTGGCGGTGAAGACGATTGGGATGACGAAGTTGAAGAGAACGGCTATGTTGCCTCTGGTTCTGCCAAAGCGAGCAAACCACGCGACGAAGAAAGCTGGGAC‑GA                                                                                                                                                                                                                                                                                              >  1:374685/1‑301`  
                    `GACCAAAGAGACC‑AAGCACATCAATCTGGTTGTGGTTGACTCAA‑‑‑‑‑AAGGTAAGAAGATGGAAGACGTTCCGATTATCGGTGGTGGCTCTAAGCTGAAAGTTAAATATTCTCTGGTTCCATACAAGTGGAACACTGCTGTAGGTGCGAGCGTTAAGCTGCAACTGGAATCCGTGATGCTGGTCGAACTGGCTACCTTTGGTGGCGGTGAAGACGATTGGGCTGACGAAGTTGAAGAGAACGGCTATGTTGCCTCTGGTTCTGCCAAAGCGAGCAAACCACGCGACGAAGAAAGCTGGGAC‑GAA                                                                                                                                                                                                                                                                                             >  1:128473/1‑301`  
                    `GACCAAAGAGACC‑AAGCACATCAATCTGGTTGTGGTTGACTCAA‑‑‑‑‑AAGGTAAGAAGATGGAAGACGTTCCGATTATCGGTGGTGGCTCTAAGCTGAAAGTTAAATATTCTCTGGTTCCATACAAGTGGAACACTGCTGTAGGTGCGAGCGCTAAGCTGCAACTGGAATCCGTGATGCTGGTCGAACTGGCTACCTTTGGTGGCGGTGAAGACGATTGGGCTGACGAAGTTGAAGAGAACGGCTATGTTGCCTCTGGTTCTGCCAAAGCGAGCAAACCACGCGACGAAGAAAGCTGGGAC‑GAA                                                                                                                                                                                                                                                                                             >  1:96021/1‑301`  
                     `ACCAAAGAGACC‑AAGCACATCAATCTGGTTGTGGTTGACTCAA‑‑‑‑‑AAGGTAAGAAGATGGAAGACGTTCCGATTATCGGTGGTGGCTCTAAGCTGAAAGTTAAATATTCTCTGGTTCCATACAAGTGGAACACTGCTGTAGGTGCGAGCGTTAAGCTGCAACTGGAATCCGTGATGCTGGTCGAACTGGCTACCTTTGGTGGCGGTGAAGACGATTGGGCTGACGAAGTTGAAGAGAACGGCTATGTTGCCTCTGGTTCTGCCACAGCGAGCAAACCACGCGACGAAGAAAGCTGGGAC‑GAAG                                                                                                                                                                                                                                                                                            >  1:150093/1‑301`  
                     `ACCAAAGAGACC‑AAGCACATCAATCTGGTTGTGGTTGACTCAA‑‑‑‑‑AAGGTAAGAAGATGGAAGACGTTCCGATTATCGGTGGTGGCTCTAAGCTGAAAGTTAAATATTCTCTGGTTCCATACAAGTGGAACACTGCTGTAGGTGCGAGCGTTAAGATGCAACTGGAGTCCGTGATGCTGGTCGAACTGGCTACCTTTGGTGGCGGTGAAGACGATTGGGCTGACGAAGTTGAAGAGAACGGCTATGTTGCCTCTGGTTCTGCCAAAGCGAGCAAACCACGCGACGAAGAAAGCTGGGAC‑GAAa                                                                                                                                                                                                                                                                                            >  1:336963/1‑300`  
                     `ACCAAAGAGACC‑AAGCACATCAATCTGGTTGTGGTTGACTAAA‑‑‑‑‑AAGGTAAGAAGATGGAAGACGTTCAGATTATCGGTGGTGGCTCTAAGCTGAAAGTTAAATATTCTCTGGTTCCATACAAGTGGAACACTGCTGTAGGTGCGAGCGTTAAGCTGCAACTGGAATCCGTGATGCTGGTCGAACTGGCTACCTTTGGTGGCGGTGAAGACGATTGGGCTGACGAAGTTGAAGAGAACGGCTATGTTGCCTCTGGTTCTGCCAAAGCGAGCAAACCCAGCGACGAAGAAAGCTGGGAC‑GAAG                                                                                                                                                                                                                                                                                            >  1:350581/1‑301`  
                      `cgtgggatcggagatgtgtataagagacagGTGGTTGACTCAA‑‑‑‑‑AAGGTAAGAAGATGGAAGACGTTCCGATTATCGGTGGTGGCTCTAAGCTGAAAGTTAAATATTCTCTGGTTCCATACAAGTGGAACACTGCTGTAGGTGCGAGCGTTAAGCTGCAACTGGAATCCGTGATGCTGGTCGAACTGGCTACCTTTGGTGGCGGTGAAGACGATTGGGCTGACGAAGTTGAAGAGAACGGCTATGTTGCCTCTGGTTCTGCCAAAGCGAGCAAACCACGCGACGAAGAAAGCTGGGAC‑GAAG                                                                                                                                                                                                                                                                                            <  1:317345/271‑1`  
                      `CCAAAGATGCC‑AAGCACATCAATCTGGGTGTGGTTGACGAAA‑‑‑‑‑AAGGTAATAAGATGGAAGACGTTCCGATTAGCGGTGGTGTCTCTAAGCTGAAAGTTAAATATTCTCTGGTTCCATACAAGTGGAACACTGCTGTAGGTGCAAGCGTTAAGCTGCAACTGGAATCCGTGATGCTGGTCGAACTGTCTACCTTTGGTGGCGGTGAAGACGATTGGTCTGACGAAGTTGAAGAGAACGGCTATGTTGCCTCTGGTTCTGCCAAAGCGAGCAAACCACGCGACGAAGAAAGCTGGGAC‑GAAGA                                                                                                                                                                                                                                                                                           <  1:215094/301‑1`  
                      `CCAAAGAGACC‑AAGCACATCAATCTGGTTGTGGTTGACTCAA‑‑‑‑‑AAGGTAAGAAGATGGAAGACGTTCCGATTATCGGTGGTGGCTCTAAGCTGAAAGTTAAATATTCTCTGGTTCCATACAAGTGGAACACTGCTGTAGTTGCGAGCGTTAAGCTGCAACTGGAATCCGTGATGCTGGTCGAACTGGCTACCTTTGGTGGCGGTGAAGACGATTGGGCTGACGAAGTTGAATAGAACGGCTATGTTGCCTCTGGTTCTGCCAAAGCGAGCAACCCACGCGACAAAGAAAGCTGGGAC‑GAAGA                                                                                                                                                                                                                                                                                           >  1:304460/1‑301`  
                      `CCAAAGAGACC‑AAGCACATCAATCTGGTTGTGGTTGACTCAA‑‑‑‑‑AAGGTAAGAAGATGGAAGACGTTCCGATTATCGGTGGTGGCTCTAAGCTGAAAGTTAAATATTCTCTGGTTCCATACAAGTGGAACACTGCTGTAGGTGCGAGCGTTAAGCTGCAACTGGAATCCGTGATGCTGGTCGAACTGGCTACCTTTGGTGGCGGTGAAGACGATTGGGCTGACGAAGTTGAAGAGAACGGCTATGTTGCCTCTGGTTCTGCCAAAGCGAGCAAACCCCGCGACGAAGAAAGCTGGGAC‑GAAGA                                                                                                                                                                                                                                                                                           >  1:331575/1‑301`  
                       `atAAGAGACCGAAGCACATGAAGTTGGTTGGGGTTGACTCAA‑‑‑‑‑AAGGTAAGAAGATGGAAGACGTGCCGATTATCGGTGGTGGCGCTAAGCTGAAAGTTAAATATTCTCTGGTTCCATACAAGTGGAACACTGCTGTAGGTGCGAGCGTTAAGCTGCAACTGGAATCCGTGATGCTGGTCGAACTGGCTACCTTTGGTGGCGGTGAAGACGATTGGGCTGACGAAGTTGAAGAGAACGGCTATGTTGCCTCTGGTTCTGCCAAAGCGAGCAAACCACGCGACGAAGAAAGCTGGGAC‑GAAGA                                                                                                                                                                                                                                                                                           <  1:263929/299‑1`  
                       `agccagGACC‑AAGCACATCAATCTGGTTGTGGTTGACTCAA‑‑‑‑‑AAGGTAAGAAGATGGAAGACGTTACGATTATCGGTGGTGGCTCTAAGCTGAAAGTTAAATATTCTCTGGTTCCATACAAGTGGAACACTGCTGTAGGTGCGAGCGTTAAGCTGCAACTGGAATCCGTGATGCTGGTCGAACTGGCTACATTTGGTGTCGGTGAAGACGATTGGGCTGACGAAGTTGAAGAGAACGGCTATGTTGCCTCTGGTTCTGCCAAAGCGAGCAAACCACGCGACGAAGAAAGCTGGGAC‑GAAGAC                                                                                                                                                                                                                                                                                          <  1:95209/295‑1`  
                       `agAAGAGACC‑AAGAACATCAATCTGGTTGTGGTTGACTCAA‑‑‑‑‑AAGGTAAGAAGATGGAAGACGTTCCGATTATCGGTGGTGGCTCTAAGCTGAAAGTTAAATATTCTCGGGTTCCATACAAGTGGAACACTGCTGTAGGTGCGAGCGTTAAGCTGCAACTGGAATCCGTGATGCTGGTCGAACTGGCTACCTTTGGTGGCGGTGAAGACGATTGGGCTGACGAAGTTGAAGAGAACGGCTATGTTGCCTCTGGTTCTGCCAAAGCGAGCAAACCACGCGACGATGAAAGCTGGGAC‑GAAGAC                                                                                                                                                                                                                                                                                          <  1:485093/299‑1`  
                       `acagGAGACC‑AAGCACATCAATCTGGTTGTGGTTGACTCAA‑‑‑‑‑AAGGTAAGAAGATGGAAGACGTTCCGATTATCGGTGGTGGCTCTAAGCTGAAAGTTAAATATTCTCTGGTTCCATACAAGTGGAACACTGCTGTAGGTGCGAGCGTTAAGCTGCAACTGGAATCCGTGATGCTGGTCGAACTGGCTACCTTTGGTGGCGGTGAAGACGATTGGGCTGACGAAGTTGAAGAGAACGGCTATGTTGCCTCTGGTTCTGCCAAAGCGAGCAAACCACGCGACGAAGAAAGCTGGGAC‑GAAGAC                                                                                                                                                                                                                                                                                          <  1:494220/297‑1`  
                       `aAAAGAGACC‑AAGCACATCAATCTGGTTGTGGTTGACTCAA‑‑‑‑‑AAGGTAAGAAGATGGAAGACGTTCCGATTATCGGTGGTGGCTCTAAGCTGAAAGTTAAATATTCTCTGGTTCCATACAAGTGGAACACTGCTGTAGGTGCGAGTGTTAAGCTGCAACTGGAATCCGTGATGCTGGTCGAACTGGCTACCTTTGGTGGCGGTGAAGACGATTGGGCTGACGAAGTTGAAGAGAACGGCTATGTTGCCTCTGGTTCTGCCAAAGCGAGCAAACCACGCGACGAAGAAAGCTGGGAC‑GAAGAC                                                                                                                                                                                                                                                                                          <  1:445092/300‑1`  
                       `CAAAGAGACC‑AAGGACATCAATCTGGTTGTGGTAGACTCAA‑‑‑‑‑AAGGTAAGAAGATGGAAGAAGTTCCGATTATCGGTGGTGGCTCTAAGCTGAAAGTTAAATATTCTCTGGTTCCATACAAGTGGAACACTGCTGTAGGTGCGAGCGTTAAGCTGCAACTGGAATCCGTGATGCTGGTCGAACTGGCTACCTTTGGTGGCGGTGAAGACGATTGGGCTGACGAAGTTGAAGAGAACGGCTATGTTGCCTCTGGTTCTGCCAAAGCGAGCAAACCACGCGACGAAGAAAGCTGGGAC‑GAAGAC                                                                                                                                                                                                                                                                                          <  1:140646/301‑1`  
                       `CAAAGAGACC‑AAGCACATCAATGTGGTTGTGGGTGACTCAA‑‑‑‑‑AAGGTAAGAAGATGGAAGACGTTCCGATTAGCGGTGGTGGCTCTAAGCGGAAAGTTAAATATTCTGTGGTTCCATACAAGTGGAACACTGCTGTAGGGGCGAGCGTTAAGCTGCAACTGGAATCCGTGATGCTGGTCGAACTGGCTACCTTTGGTGGCGGTTAAGACGATTGGGCTGACGAAGTTGAAGAGAACGGCTATGTTGCCTCTGGTTCTGCCAAAGCGAGCAAACCACGCGACGAAGAAAGCTGGGAC‑GAAGAC                                                                                                                                                                                                                                                                                          <  1:303285/301‑1`  
                       `CAAAGAGACC‑AAGCACATCAATCTTGTTGTGGTTGACTCAA‑‑‑‑‑AAGGTAAGAAGATGGAAGACGTTCCGATTATCGGTGGTGGCTCTAAGCTGAAAGTTAAATATTCTCTGGTTCCATACAAGTGGAACACTGCTGTAGGTGCGAGCGTTAAGCTGCAACTGGAATCCGTGATGCTGGTCGAACTGGCTACCTTTGGTGGCGGTGAAGACGATTGGGCTGACGAAGTTGAAGAGAACGGCTATGTTGCCTCTGGTTCTGCCAAAGCGAGCAAACCACGCGACGAAGAAAGCTGGGAC‑GAAGAC                                                                                                                                                                                                                                                                                          <  1:225724/301‑1`  
                       `CAAAGAGACC‑AAGCACATCAATCTGGTTGTGGTTGACTCAA‑‑‑‑‑AAGGTAAGAAGATGGAAGACGTTCCGATTATCGGTGGTGGCTCTAAGCTGAAAGTTAAATATTCTCTGGTTCCATACAAGTGGAACACTGCTGTAGGTGCGAGCGTTAAGCTGCAACTGGAATCCGTGATGCTGGTCGAACTGGCTACCTTTGGTGGCGGTGAAGACGATTGGGCTGACGAAGTTGAAGAGAACGGCTATGTTGCCTCTGGTTCTGCCAAAGCGAGCAAACCACGCGACGAAGAAAGCTGGGAC‑GAAGAC                                                                                                                                                                                                                                                                                          <  1:402949/301‑1`  
                        `gtcggagatgtgtataagagacagGGTTGTGGTTGACTCAA‑‑‑‑‑AAGGTAAGAAGATGGAAGACGTTCCGATTATCGGTGGTGGCTCGAAGCTGAAAGTTAAATATTCTCTGGTTCCATACAAGTGGAACACTGCTGTAGGTGCGAGCGTTAAGCTGCAACTGGAATCCGTGATGCTGGTCGAACTGGCTACCTTTGGTGGCGGTGAAGACGATTGGGCTGACGAAGTTGAAGAGAACGGCTATGTTGCCTCTGGTTCTGCCAAAGCGAGCAAACCACGCGACGAAGAAAGCTGGGAC‑GAAGAC                                                                                                                                                                                                                                                                                          <  1:65544/277‑1`  
                          `atgtgtataagagacagAATCTGGTTGTGGTTGACTCAA‑‑‑‑‑AAGGTAAGAAGATGGAAGACGTTCCGATTATCGGTGGTGGCTCTAAGCTGAAAGTTAAATATTCTCTGGTTCCATACAAGTGGAACACTGCTGTAGGTGCGAGCGTTAAGCTGCAACTGGAATCCGTGATGCTGGTCGAACTGGCTACCTTTGGTGGCGGTGAAGACGATTGGGATGACGAAGTTGCAGAGAACGGCTATGTTGCCTCTGGTTCTGCAAAAGCGAGCAAACCACGCGACGAAGAAAGCTGGGAC‑GAAGACGA                                                                                                                                                                                                                                                                                        <  1:165585/284‑1`  
                          `AGAGACC‑AAGCACATCAATCTGGTTGTTGTTGACTCAA‑‑‑‑‑AATGTAAGAAGATGGAAGACGGTCCGATTATCGGTGGTGGCTCTAAGCTGAAAGTTAAATATTCTCTGGTTCCATACAAGTGGAACACTGCTGTAGGTGCGAGCGTTAAGCTGCAACTGGAATCCGTGATGCTGGTCGAACTGGCTACCTTTGGTGGCGGTGAAGACGATTGGGCTGACGAAGTTGAAGAGAACGGCTATGTTGCCTCTGGTTCTGCCAAAGCGAGCAAACCACGCGACGAAGAAAGCTGGGAC‑GAAGACGAC                                                                                                                                                                                                                                                                                       <  1:256194/301‑1`  
                           `GAGACC‑AAGCACATCAATCTGGTTGTGGTTGACTCAA‑‑‑‑‑AAGGTAAGAAGATGGAAGACGTTCCGATTATCGGTGGTGGCTCTAAGCTGAAAGTTAAATATTCTCTGGTTCCATACAAGTGGAACACTGCTGTAGGTGCGAGCGTTAAGCTGCAACTGGAATCCGTGATGCTGGTCGAACTGGCTACCTTTGGTGGCGGTGAAGACGATTGGGCTGACGAAGTTGAAGAGAACGGCTATGTTGCCTCTGGTTCTGCCAATGCGAGCAAACCACGCGACGAAGAAAGCTGGGAC‑GAAGACGACG                                                                                                                                                                                                                                                                                      >  1:248622/1‑301`  
                           `GAGACC‑AAGCACATCAATCTGGTTGTGGTTGACTCAA‑‑‑‑‑AAGGTAAGAAGATGGAAGACGTTCCGATTATCGGTGGTGGCTCTAAGCTGAAAGTTAAATATTCTCTGGTTCCATACAAGTGGAACACTGCTGTAGGTGCGAGCGTTAAGCTGCAACTGGAATCCGTGATGCTGGTCGAACTGGCTACCTTTGGTGGCGGTGAAGACGATTGGGCTGACGAAGTTGAAGAGAACGGCTATGTTGCCTCTGGTTCTGCCAAAGCGAGCAAACCACGCGACGAAGAAAGCctgtctcttatacaca                                                                                                                                                                                                                                                                                       >  1:252192/1‑285`  
                           `GAGACC‑AAGCACATCAATCTGGTTGTGGTTGACTCAA‑‑‑‑‑AAGGTAAGAAGATGGAAGACGTTCCGATTATCGGTGGTGGCTCTAAGCTGAAAGTTAAATATTCTCTGGTTCCATACAAGTGGAACACTGCTGTAGGTGCGAGCGTTAAGCTGCAACTGGAATCCGTGATGCTGGTCGAACTGGCTACCTTTGGTGGCGGTGAAGACGATTGGGCTGACGAAGTTGAAGAGAACGGCTATGTTGCCTCTGGTTCTGCCAAAGCGAGCAAACCACGCGACGAAGAAAGCTGGGAC‑GAAGACGACG                                                                                                                                                                                                                                                                                      >  1:311391/1‑301`  
                           `GAGACC‑AAGCACATCAATCTGGTTGTGGTTGACTCAA‑‑‑‑‑AAGGTAAGAAGATGGAAGACGTTCCGATTATCGGTGGTGGCTCTAAGCTGAAAGTTAAATATTCTCTGGTTCCATACAAGTGGAACACTGCTGTAGGTGCGAGCGTTAAGCTGCAACTGGAATCCGTGATGCTGGTCGAACTGGCTACCTTTGGTGGCGGTGAAGACGATTGGGCTGACGAAGTTGAAGAGAACGGCTATGTTGCCTCTGGTTCTGCCAAAGCGAGCAAACCACGCGACGAAGAAAGCTGGGAC‑GAAGACGACG                                                                                                                                                                                                                                                                                      >  1:367040/1‑301`  
                           `GAGACC‑AAGCACATCAATCTGGTTGTGGTTGACTCAA‑‑‑‑‑AAGGTAAGAAGATGGAAGACGTTCCGATTATCAGTGGTGGCTCTAAGCTGAAAGTTAAATATTCTCTGGTTCCATACAAGTGGAACACTGCTGTAGGTGCGAGCGTTAAGCTGCAACTGGAATCCGTGATGCTGGTCGAACTGGCTACCTTTGGTGGCGGTGAAGACGATTGGGCTGACGAAGTTGAAGAGAACGGCTATGTTGCCTCTGGTTCTGCCAAAGCGAGCAAACCACGCGACGAAGAAAGCTGGGAC‑GAAGACGACG                                                                                                                                                                                                                                                                                      >  1:102111/1‑301`  
                            `AGACC‑AAGCACATCAATCTGGTTGTGGTTGACTCAA‑‑‑‑‑AAGGTAAGAAGATGGAAGACGTTCCGATTATCGGTGGTGGCTCTAAGCTGAAAGTTAAATATTCTCTGGTTCCATACAAGTGGAACACTGCTGTAGGTGCGAGCGTTAAGCTGCAACTGGAATCCGTGATGCTGGTCGAACTGGCTACCTTTGGTGGCGGTGAAGACGATTGGGCTGACGAAGTTGAAGAGAACGGCTATGTTGCCTCTGGTTCTGCCAAAGCGAGCAAACCACGCGACGAAGAAAGCTGGGAA‑GAAGACGACGA                                                                                                                                                                                                                                                                                     >  1:158412/1‑301`  
                            `AGACC‑AAGCACATCAATCTGGTTGTGGTTGACTCAA‑‑‑‑‑AAGGTAAGAAGATGGAAGACGTTCCGATTATCGGTGGTGACTCTAAGCTGAAAGTTAAATATTCTCTGGTTCCATACAAGTGGAACACTGCTGTAGGTGCGAGCGTTAAGCTGCAACTGGAATCCGTGATGCTGGTCGAACTGGCTACCTTTGGTGGCGGTGAAGACGATTGGGCTGACGAAGTTGAAGAGAACGGCTAAGTTGCCTCTGGTTCTGCCAAAGCGAGCAAACCACGCGACGAAGAAAGCTGGGAC‑GAAGACGATGA                                                                                                                                                                                                                                                                                     >  1:26720/1‑301`  
                            `AGACC‑AAGCACATCAATCT‑GTTGTGGTTGACTCAA‑‑‑‑‑AAGGTAAGAAGATGGAAGACGTTCCGATTATCGGTGGTGGCTCTAAGCTGAAAGTTAAATATTCTCTGGTTCCATACAAGTGGAACACTGCTGTAGGTGCGAGCGTTAAGCTGCAAATGGAATCCGTGATGCTGGTGGAACTGGCTACCTTTGGTGGCGGTGAAGACGATTGGGCTGACGAAGTTGAAGAGAAGGGCTATGTTACCTCTGGTTCTGCCAAAGCGAGCAAACAACGAGACGAAGAAAGCAGGGAC‑GAAGACGACGAg                                                                                                                                                                                                                                                                                    >  1:384546/1‑300`  
                             `GACC‑AAGCACATCAATCTGGTTGTGGTTGACTCAA‑‑‑‑‑AAGGTAAGAAGATGGAAGACGTTCCGATTATCGGTGGTGGCTCTAAGCTGAAAGTTAAATATTCTCTGGTTCCATACAAGTGGAACACTGCTGTAGGTGCGAGCGTTAAGCTGCAACTGGAATCCGTGATTCTGGTCGAACTGGCTACCTTTGGTGGCGGTGAAGACGATTGGGCTGACGAAGTTTAAGAGAACGGCTATGTTGCCTCTGGTTCTGCCAAAGCGAGCAAACCACGCGACGAAGAAAGCTGGGAC‑GAAGACGACGAA                                                                                                                                                                                                                                                                                    >  1:404153/1‑301`  
                             `GACC‑AAGCACATCAATCTGGTTGTGGTTGACTCAA‑‑‑‑‑AAGGTAAGAAGATGGAAGACGTTCCGATTATCGGTGGTGGCTCTAAGCTGAAAGTTAAATATTCTCTGGTTCCATACAAGTGGAACACTGCTGTAGGTGCGAGCGTTAAGCTGCAACTGGAATCCGTGATGCTGGTCGAACTGGCTACCTTTGGTGGCGGTGAAGACGATTGGGCTGACGAAGTTGAAGAGAACGGCTATGTTGCCTCTGGTTCTGCCAAAGCGAGCAAACCACGCGACGAAGAAAGCTGGGAC‑GACGACGACGAA                                                                                                                                                                                                                                                                                    >  1:207395/1‑301`  
                             `GACC‑AAGCACATCAATCTGGTTGTGGTTGACTCAA‑‑‑‑‑AAGGTAAGAAGATGGAAGACGTTCCGATTATCGGTGGTGGCTCTAAGCTGAAAGTTAAATATTCTCTGGTTCCATACAAGTGGAACACTGCTGTAGGTGCGAGCGTTAAGCTGCAACTGGAATCCGTGATGCTGGTCGAACTGGCTACCTTTGGTGGCGGTGAAGACGATTGGGCTGACGAAGTTGAAGAGAACGGCTATGTTGCCTCTGGTTCTGCCAAAGCGAGCAAACCACGCGACGAAGAAAGCTGGGAC‑GAAGACGACGAA                                                                                                                                                                                                                                                                                    >  1:489655/1‑301`  
                             `GACC‑AAGCACATCAATCTGGTTGTGGTTGACTCAA‑‑‑‑‑AAGGTAAGAAGATGGAAGACGTTCCGATTATCGGTGGTGGCTCTAAGCTGAAAGTTAAATATTCTCTGGTTCCATACAAGTGGAACACTGCTGTAGGTGCGAGCGTTAAGCTGCAACTGGAATCCGTGATGCTGGTCGAACTGGCTACCTTTGGTGGCGGTGAAGACGATTGGGCTGACGAAGTTGAAGAGAACGGCTATGTTGCCTCTGGTTCTGCCAAAGCGAGCAAACCACGCGACGAAGAAAGCTGGGAC‑GAAGACGACGAA                                                                                                                                                                                                                                                                                    >  1:181727/1‑301`  
                             `GACC‑AAGCACATCAATCTGGTTGTGGTTGACTCAA‑‑‑‑‑AAGGTAAGAAGATGGAAGACGTTCCGATTATCGGTGGTGGCTCTAAGCTGAAAGTTAAATATTCTCTGGTTCCATACAAGTGGAACACTGCTGTAGGTGCGAGCGTTAAGCTGCAACTGGAATCCGTGATGCTGGTCGAACTGGCTACCTTTGGTGGCGGTGAAGACGATAGGGCTGACGAAGTTGAAGAGAACGGCTCTGTTGCCTCTGGTTCTGCCAAAGCGAGCAAACCACGCGACGAAGAAAGCTGGGAC‑GAAGACGACGAA                                                                                                                                                                                                                                                                                    >  1:79491/1‑301`  
                             `GACC‑AAGCACATCAATCTGGTTGTGGTTGACTCAA‑‑‑‑‑AAGGTAAGAAGATGGAAGACGTTCCGATTATCGGTGGTGGCTCTAAGCTGAAAGTTAAATATTCTCTGGTTCAATACAAGTGGAACACTGCTGTAGGTGCGAGCGTTAAGATGCAACTGGAATCCGTGATGCTGGTCGAACTGGCTACCTTTGGTGGCGGTGAAGACGATTGGGCTGACGAAGTTGAAGAGAACGGCTATGTTGCCTCTGGTTCTGCCAAAGCGAGCAAACCACGCGACGAAGAAAGCTGGGAC‑GAAGACGACGAA                                                                                                                                                                                                                                                                                    >  1:106771/1‑301`  
                              `ACC‑AAGCACATCAATCTGGTTGTGGTTGACTCAA‑‑‑‑‑AAGGTAAGAAGATGGAAGACGTTCCGATTATCGGTGGTGGCTCTAATCTGAAAGTTAAATATTCTCTGGTTCCATACAAGTGGAACACTGCTGTAGGTGCGAGCGTTAAGCTGCAACTGGAATCCGTGATGCTGGTCGAACTGGCTACCTTTGGTGGCGGTGAAGACTATTGGGCTGACGAAGTTGAAGAGAACGGCTATGTTGCCTCTGGTTCTGCCAAAGCGAGCAAACCACGCGACGAAGAAAGCTGGGAC‑GAAGACGACGAAG                                                                                                                                                                                                                                                                                   <  1:220246/301‑1`  
                              `ACC‑AAGCACATCAAGCTGGTTGTGGTTTACTCAA‑‑‑‑‑AAGGTAAGAAGATGGAATACGTTCCGATTATCGGTGGGGGCTCTAAGCTGAAAGTTAAATATTCTCTGGTTCCATACAAGTGGAACACTGCTGTAGGTGCGATCGTTAAGCTGCAACTGGAATCCGTGATGCTGGTCGAACTGGCTACCTTTGGTGGCGGTGAAGACGATTGGGCTGACGAAGTTGAAGAGAACGGCTATGTTGCCTCTGGTTCTACCAAAGCGAGCAAACCACGCGACGAAGAAAGCTGGGAC‑GAAGACGACGAAG                                                                                                                                                                                                                                                                                   <  1:209581/301‑1`  
                               `gagacagACATCAATCGGGTTGTGGTTGACTCAA‑‑‑‑‑AAGGTAAGAAGATGGAAGACGTTCCGATTATCGGTGGTGGCTCTAAGCTGAAAGTTAAATATTCTCTGGTTCCATACAAGTGGAACACTGCTGTAGGTGCGAGCGTTAAGCTGCAACTGGAATCCGTGATGCTGGTCGAACTGGCTACCTTTGGTGGCGGTGAAGACGATTGGGCTGACGAAGTTGAAGAGAACGGCTATGTTGCCTCTGGTTCTGCCAAAGCGAGCAAACCACGCGACGAAGAAAGCTGGGAC‑GAAGACGACGAAG                                                                                                                                                                                                                                                                                   <  1:426059/294‑1`  
                               `accaagacaATCAATCTGGTTGGGGTTGACTAAA‑‑‑‑‑AAGGTAAGAAGATGGAAGACGTTACGATTAGCGGTGGTGGTTCTAAGCTGAAAGTTAAATATTATCTGGTTCCATACAAGTGGAACACTGCTGTAGGTGCGAGCGTTAAGCTGCAACTGGAATCCGTGATGCTTGTCGAACTGGCTACCTTTGGTGGCGGTGAAGACGATTGGGCTGACGAAGTTGAAGAGAACGGCTATGTTGCCTCTGGTTCTGCCAAAGCGAGCAAACCACGCGACGAAGAAAGCTGGGAC‑GAAGACGACGAAG                                                                                                                                                                                                                                                                                   <  1:259053/292‑1`  
                               `CC‑AAGCACATCAATCTGGTTGTGGTTGACTCAA‑‑‑‑‑AAGGTAAGAAGATGGAAGACGTTCCGATTATCGGTGGTGGCTCTAAGCTGAAAGTTAAATATTCTCTGGTTCCATACAAGTGGAACACTGCTGTAGGTGCGAGCGTTAAGCTGCAACTGGAATCCGTGATGCTGGTCGAACTGGCTACCTTTGGTGGCGGTGAAGACGATTGGGCTGACGAAGTTGAAGAGAACGGCTATGTTGCCTCTGGTTCTGCCAAAGCGAGCAAACCACGCGACGAAGAAAGCTGGGAC‑GAAGACGACGAAGA                                                                                                                                                                                                                                                                                  >  1:507805/1‑301`  
                               `CC‑AAGCACATCAATCTGGTTGTGGTTGACTCAA‑‑‑‑‑AAGGTAAGAAGATGGAAGACGTTCCGATTATCGGTGGTGGCTCTAAGCTGAAAGTTAAATATTCTCTGGTTCCATACAAGTGGAACACTGCTGTAGGTGCGAGCGTTAAGCTGCAACTGGAATCCGTGATGCTGGTCGAACTGGCTACCTTTGGTGGCGGTGAAGACGATTGGGCTGACGAAGTTGAAGAGAACGGCTATGTTGCCTCTGGTTCTGCCAAAGCGAGCAAACCACGCGACGAAGAAAGCTGGAAC‑GAAGACGACGAAGg                                                                                                                                                                                                                                                                                  >  1:493645/1‑300`  
                               `CC‑AAGCACATCAATCTGGTTGTAGTTGACTCAA‑‑‑‑‑AAGGTAAGAAGATGGAAGACGTTCCGATTATCGGTGGTGGCTCTAAGCTGAAAGTTAAATATTCTCTGGTTCCACACAAGTGGAACACTGCTGTAGGTGCGAGCGTTAAGCTGTAACTGGAATCCGTGATGCTGGTCGAACTGGCTACCTTTGGTGGCGGTGAAGACGATTGGGCTGACGAAGTTGAAGAGAACGGCTATGTTGCCTCTGGTTCTGCCAAAGCGAGCAAACCACGCGACGAAGAAAGCTGGGAC‑GAAGACGACAAAGA                                                                                                                                                                                                                                                                                  >  1:116419/1‑301`  
                                `ataagggacagAATATGGTTGTGGTTGACTCAA‑‑‑‑‑AAGGTAAGAAGATGGAAGACGTTCCGATTATCGTTGGTGGCTCTAAGCTGAAAGTTAAATATTCTCTGGTTCCATACAAGTGGAACACTGCTGTAGGTGCGAGCTTTAAGCTGCAACTGGAATCCGTGGTGCTGGTCGAACTGGCTACCTTTGGTGGCGGTGAAGACGATTGGGCTGACGAAGTTGAAGAGAACGGCTATGTTGCCTCTGGTTATGCCAAAGCGAGCAAACCACGCGACGAAGAAAGCTGGGAC‑GAAGACGACGAAGA                                                                                                                                                                                                                                                                                  <  1:454157/290‑1`  
                                `aaAAGCACATCAATCTGGTTGTGGTTGACTCAA‑‑‑‑‑AAGGTAAGAAGATGGAAGACGTTCCGATTATCGGTGGTGGCTCTAAGCTGAAAGTTAAATATTCTCTGGTTCCATACAAGTGGAACACTGCTGTAGGTGCGGGCGTTAAGCTGCAACTGGAATCCGTGATGCTGGTCGAACTGGCTACCTTTGGTGGCGGTGAAGACGATTGGGCTGACGAAGTTGAAGAGAACGGCTATGTTGCCTCTGGTTCTGCCAAAGCGAGCAAACCACGCGACGAAGAAAGCTGGGAC‑GAAGACGACGAAGA                                                                                                                                                                                                                                                                                  <  1:92202/299‑1`  
                                  `AAGCACATCAATCTGGTTGTGGTTGACTCAA‑‑‑‑‑AAGGTAAGAAGATGGAAGACGTTCCGATTATCGGTGGTGGCTCTAAGCTGAAAGTTAAATATTCTCTGGTTCCATACAAGTGGAACACTGCTGTAGGTGCGAGCGTTAAGCTGCAACTGGAATCCGTGATGCTGGTCGAACTGGCTACCTTTGGTGGCGGTGAAGACGATTGGGCTGACGCAGTTGAAGAGAACGGCTATGTTGCCTCTGGTTCTGCCAAAGCGAGCAAACCACGCGACGAAGAAAGCTGGGAC‑GAAGACctgtctcttt                                                                                                                                                                                                                                                                                >  1:295733/1‑291`  
                                  `AAGCACATCAATCTGGTTGTGGTTGACTCAA‑‑‑‑‑AAGGTAAGAAGATGGAAGACGTTCCGATTATCGGTGGTGGCTCTAAGCTGAAAGTTAAATATTCTCTGGTTCCATACAAGTGGAACACTGCTGTAGGTGCGAGCGTTAAGCTGCAACTGGAATCCGTGATGCTGGTCGAACTGGCTACCTTTGGTGGCGGTGAAGACGATTGGGCTGACGAAGTTGAAGAGAACGGCTATGTTGCCTCTGGTTCTGCCAAAGCGAGCAAACCACGCGACGAAGAAAGCTGGGAC‑GAAGACGACGAAGAtt                                                                                                                                                                                                                                                                                >  1:39006/1‑299`  
                                    `GCACATCAATCTGGTTGTGGTTGACTCAA‑‑‑‑‑AAGGTAAGAAGATGGAAGACGTTCCGATTATCGGTGGTGGCTCTAAGCTGAAAGTTAAATATTCTCTGGTTCCATACAAGTGGAACACTGCTGTAGGTGCGATCGTTAAGCTGCAACTGGAATCCGTGATGCTGGTCGAACTGGCTACCTTTGGTGGCGGTGAAGACGATTGGGCTGACGAAGTTGAAGAGAACGGCTATGTTGCCTCTGGTTCTGCCAAAGCGAGCAAACCACGCGACGAAGAAGGCTGGGAC‑GAAGACGACGAAGActgt                                                                                                                                                                                                                                                                              >  1:68451/1‑297`  
                                      `ACATCAATCTGGTTGTGGTTGACTCAA‑‑‑‑‑AAGGTAAGAAGATGGAAGACGTTCCGATTATCGGTGGTGGCTCTAAGCTGAAAGTTAAATATTCTCTGGTTCCATACAAGTGGAACACTTCTGTAGGTGCGAGCGTTAAGCTGCAACTGGAATCCGTGATGCTGGTCGAACTGGCTACCTCTGGTGGCGGTGAAGCCGATTGGGCTGACGAAGTTGAAGAGAACGGCTATGTTACCTCTGGTTCTGCCCAAGCGAGCAACACACGCGACGCAGAACGCTGGGCC‑GAAGCCGACGAAGActgtct                                                                                                                                                                                                                                                                            >  1:416417/1‑295`  
                                      `ACATCAATCTGGTTGTGGTTGACTCAA‑‑‑‑‑AAGGTAAGAAGATGGAAGACGTTCCGATTATCGGTGGTGGCTCTAAGCTGAAAGTTAAATATTCTCTGGTTCCATACAAGTGGAACACTGCTGTAGGTGCGAGCGTTAAGCTGCAACTGGAATCCGTGATGCTGGTCGAACTGGCTACTTTTGGTGGCGGTGAAGACGATTGGGCTGACGAAGATAAAGAGAACGGCTATGTTGCCTCTGGTTCTGCCAAAGCGAGCAAACCACGCGACGAAGAAAGCTGGGAC‑GAAGACGACGAAGctgtctc                                                                                                                                                                                                                                                                            >  1:498514/1‑294`  
                                      `ACATCAATCTGGTTGTGGTTGACTCAA‑‑‑‑‑AAGGTAAGAAGATGGAAGACGTTCCGATTATCGGTGGTGGCTCTAAGCTGAAAGTTAAATATTCTCTGGTTCCATACAAGTGGAACACTGCTGTAGGTGCGAGCGTTAAGCTGCAACTGGAATCCGTGATGCTGGTCGAACTGGCTACCTTTGGTGGCGGTGAAGACGATTGGGCTTACGAAGTTGAAGAGAACAGCTATGTTGCCTCTGGTTCTGCCAAAGCGAGCAAACCACGTGACGAAGAAAGCTGGGAC‑GAAGACGACGAAGctgtctc                                                                                                                                                                                                                                                                            >  1:120208/1‑294`  
                                       `cacCAATCTGGTTGTGGTTGACTCAA‑‑‑‑‑AAGGTAAGAAGATGGAAGACGTTCCGATTATCGGTGGTGGCTCTAAGCTGAAAGTTAAATATTCTCTGGTTCCATACAAGTGGAACACTGCTGTAGGTGCGAGCGTTAAGCTGCAACTGGAATCCGTGATGCTGGTCAAACTGGCTACCTTTGGTGGCGGTGAAGACGATTGGG‑TGACGCAGTTGAAGGGAAACGCTATGTTTCCTTCGGTTCTGCCAAAGCGAGGCAACCACCCCACGAAAAAAGCTGTGACGGAAGA‑GAAGAAGAAtacgagg                                                                                                                                                                                                                                                                          >  1:255735/4‑294`  
                                       `CATCAATCTGGTTGTGGTTGACTCAA‑‑‑‑‑AAGGTAAGAAGATGGAAGACGTTCCGATTATCGGTGGTGGCTCTAAGCTGAAAGTTAAATATTCTCTGGTTCCATACAAGTGGAACACTGCTGTAGGTGCGAGCGTTAAGCTGCAACTGGAATCCGTGATGCTGGTCGAACTGGCTACCTTTGGTGGCGGTGAAGACGATTGGGCTGACGAAGTTGAAGAGAACGGCTATGTTTCCTCTGGTTCTGCCAAAGCGAGCAAACCACGCGACGAAGAAAGCTGGGAC‑GAAGACGACGAAGctgtctct                                                                                                                                                                                                                                                                           >  1:195238/1‑293`  
                                       `CATCAATCTGGTTGTGGTTGACTCAA‑‑‑‑‑AAGGTAAGAAGATGGAAGACGTTCCGATTATCGGTGGTGGCTCTAAGCTGAAAGTTAAATATTCTCTGGTTCCATACAAGTGGAACACTGCTGTAGGTGCGAGCGTTAAGCTGCAACTGGAATCCGTGATGCTGGTCGAACTGGCTACCTTTGGTGGCGGTGAAGACGATTGGGCTGACGAAGTTGAAGAGAACGGCTATGTTGCCTCTGGTTCTGCCAAAGCGAGCAAACCACGCGACGAAGAAAGCTGGGAC‑GAAGACGACGAAGctgtctct                                                                                                                                                                                                                                                                           >  1:114698/1‑293`  
                                       `CATCAATCTGGTTGTGGTTGACTCAA‑‑‑‑‑AAGGTAAGAAGATGGAAGACGTTCCGATTATCGGTGGTGGCTCTAAGCTGAAAGCTAAATATTCTCTGGTTCCATACAAGTGGAACACTGCTGTAGGTGCGAGCGTTCAGCTGCAACTGGAATCCGTGATGCTGGTCGAACTGGCTACCTTTGGTGGCGGTGAAGACGATTGGGCGGACGAAGTTGAAGAGAACGGCAATGTTGACTCTGGGTCTGCCAAAGCGAGCAAACCACGCGACGACGAAAGCGGGGAC‑GAAGACtgtatattatacaca                                                                                                                                                                                                                                                                           >  1:293184/1‑286`  
                                            `ATCTGGTTGTGGTTGACTCAA‑‑‑‑‑AAGGTAAGAAGATGGAAGACGTTCCGATTATCGGTGGTGGCTCTAAGCTGAAAGTTAAATATTCTCTGGTTCCATACAAGTGGAACACTGCTGTAGGTGCGAGCGTTAAGCTGCAACTGGAATCCGTGATGCTGGTCGAACTGGCTACCTTTGGTGGCGGTGAAGACGATTGGGCTGACGAAGTTGAAGAGAACGGCTATGTTGCCTCTGGTTCTGCCAAAGCGAGCAAACCACGCGACGAAGAAAGCTGGGAC‑GAAGACGACctgtctcttatccccat                                                                                                                                                                                                                                                                      >  1:281663/1‑284`  
                                              `CTGGTTGTGGTTGACTCAA‑‑‑‑‑AAGGTAAGAAGATGGAAGACGTTACAATTATCGGTGGTGGCTCTACGCTGAAAGTTAAATATTCTCTGGTTCCATACAAGTGGCACACTGATGTAGGTGCGAGCGTTCAGCTGCAACTGGAATCCGTGATGCTGTTCGAACTTGCTACCTATGGTGGCGGTGCAGACGATTGTGCTGACGAAGTTGAAGAGATCGGCTTTGTTTCCTCTGGTTCTGCCAAAGCGAGCAAACCACGCGACGCCTCACGCCTGGAC‑GAAGCCGACGAcgcctgtctcttataca                                                                                                                                                                                                                                                                    >  1:324634/1‑284`  
                                                            `CTCAA‑‑‑‑‑AATGTAAGAAGATGGAAGACGTTCCGATTATCGGTGGTGGCTCTAAGCTGAAAGTTAAATATTCTCTGGTTCCATACAAGTGGAACACTGCTGTAGGTGCGAGCGTTAAGCTGCAACTGGAATCCGTGATGCTGGTCGAACTGGCTACCTTTGGTGGCGGTGAAGACGATTGGGCTGACGAAGTTGAAGAGAACGGCTATGTTGCCTCTGGTTCTGCCAAGGCGAGCAAACCACGCGACGAAGAAAGCTGGGAC‑GAAGACGACGAActgtctcttatacccatctccgagcccacg                                                                                                                                                                                                                                                      >  1:456823/1‑271`  
                                                                                                                                                                                                                                                                                                                     `GAAGAAAGCTGGGAC‑GAAGACGACGAAGAAAGCTGGGACGAAGACGACGAAGAGTCCGAGGAAGCAGACGAAGACGGAGACTTCTAAGTGGAACTGCGGGAGAAAATCCTTGAGCGAATCAAGGTGACTTCCTCTGGGTGTTGGGAGTGGCAGGTCGCTACGAACAATAAAGGGTACGGGCAGGTGTGGTGCAGCAATACCGGAAAGGTTGTCTACTGTCATCGCGTAATGTCTAATGCTCCGAGAGGTTCTACCGTCCTGCACTCCTGTTATAATCCATTATGTTGTAACCCctgtctct  >  1:34648/1‑293`  

                                                                                                                                                                                                                                                                                                                 `|||||||||||||||||||||||||`                                                                                                                                                                                                                                                                                           
`GCGTCTTTCCAAGACAAGAAGACCAAAGAGACC‑AAGCACATCAATCTGGTTGTGGTTGACTCAA‑‑‑‑‑AAGGTAAGAAGATGGAAGACGTTCCGATTATCGGTGGTGGCTCTAAGCTGAAAGTTAAATATTCTCTGGTTCCATACAAGTGGAACACTGCTGTAGGTGCGAGCGTTAAGCTGCAACTGGAATCCGTGATGCTGGTCGAACTGGCTACCTTTGGTGGCGGTGAAGACGATTGGGCTGACGAAGTTGAAGAGAACGGCTATGTTGCCTCTGGTTCTGCCAAAGCGAGCAAACCACG‑‑‑‑‑‑‑‑‑‑‑‑‑‑‑‑‑‑‑‑‑‑‑‑‑‑‑‑‑‑‑‑‑‑‑‑‑‑‑‑‑‑‑‑‑‑‑‑‑‑‑‑‑‑‑‑‑‑‑‑‑‑‑‑‑‑‑‑‑‑‑‑‑‑‑‑‑‑‑‑‑‑‑‑‑‑‑‑‑‑‑‑‑‑‑‑‑‑‑‑‑‑‑‑‑‑‑‑‑‑‑‑‑‑‑‑‑‑‑‑‑‑‑‑‑‑‑‑‑‑‑‑‑‑‑‑‑‑‑‑‑‑‑‑‑‑‑‑‑‑‑‑‑‑‑‑‑‑‑‑‑‑‑‑‑‑‑‑‑‑‑‑‑‑‑‑‑‑‑‑‑‑‑‑‑‑‑‑‑‑‑‑‑‑‑‑‑‑‑‑‑‑‑‑‑‑‑‑‑‑‑‑‑‑‑‑‑‑‑‑‑‑‑‑‑‑‑‑‑‑‑‑‑‑‑‑‑‑‑‑‑‑‑‑‑‑‑‑‑‑‑‑‑‑‑‑‑‑‑‑‑‑‑‑‑‑‑‑‑‑‑‑‑‑‑‑‑‑‑‑‑‑‑‑‑‑‑‑‑‑‑‑‑‑‑‑‑‑‑‑‑‑‑‑‑‑  >  NC_001604.1/9491‑9789`  
`‑‑‑‑‑‑‑‑‑‑‑‑‑‑‑‑‑‑‑‑‑‑‑‑‑‑‑‑‑‑‑‑‑‑‑‑‑‑‑‑‑‑‑‑‑‑‑‑‑‑‑‑‑‑‑‑‑‑‑‑‑‑‑‑‑‑‑‑‑‑‑‑‑‑‑‑‑‑‑‑‑‑‑‑‑‑‑‑‑‑‑‑‑‑‑‑‑‑‑‑‑‑‑‑‑‑‑‑‑‑‑‑‑‑‑‑‑‑‑‑‑‑‑‑‑‑‑‑‑‑‑‑‑‑‑‑‑‑‑‑‑‑‑‑‑‑‑‑‑‑‑‑‑‑‑‑‑‑‑‑‑‑‑‑‑‑‑‑‑‑‑‑‑‑‑‑‑‑‑‑‑‑‑‑‑‑‑‑‑‑‑‑‑‑‑‑‑‑‑‑‑‑‑‑‑‑‑‑‑‑‑‑‑‑‑‑‑‑‑‑‑‑‑‑‑‑‑‑‑‑‑‑‑‑‑‑‑‑‑‑‑‑‑‑‑‑‑‑‑‑‑‑‑‑‑‑‑‑‑‑‑‑‑‑‑‑‑‑‑‑‑‑‑‑‑‑‑‑‑‑‑‑‑‑‑‑‑‑‑‑‑‑‑‑‑‑‑‑‑‑‑‑‑‑‑‑‑‑‑‑‑‑‑‑‑‑‑‑‑‑‑‑‑‑‑‑‑‑‑‑CGACGAAGAAAGCTGGGACGAAGACGACGAAGAGTCCGAGGAAGCAGACGAAGACGGAGACTTCTAAGTGGAACTGCGGGAGAAAATCCTTGAGCGAATCAAGGTGACTTCCTCTGGGTGTTGGGAGTGGCAGGGCGCTACGAACAATAAAGGGTACGGGCAGGTGTGGTGCAGCAATACCGGAAAGGTTGTCTACTGTCATCGCGTAATGTCTAATGCTCCGAAAGGTTCTACCGTCCTGCACTCCTGTGATAATCCATTATGTTGTAACCCTGAACACC  >  NC_001604.1/9790‑10070`

  

Alignment Legend

Aligned base mismatch/match (shaded by quality score): `ATCG/ATCG` `< 3 ≤` `ATCG/ATCG` `< 11 ≤` `ATCG/ATCG` `< 22 ≤` `ATCG/ATCG` `< 34 ≤` `ATCG/ATCG` `< 38 ≤` `ATCG/ATCG`

Unaligned base: `atcg`    Masked matching base: `atcg`    Alignment gap: `‑`    Deleted base: `‑`

Reads not counted as support for junction

read\_name Not counted due to insufficient overlap past the breakpoint.

read\_name Not counted due to not crossing MOB target site duplication.
